# Vitrification with Dimethyl Sulfoxide Induces Transcriptomic Alteration of Gene and Transposable Element Expression in Immature Human Oocytes

**DOI:** 10.3390/genes14061232

**Published:** 2023-06-08

**Authors:** Ashley Wiltshire, Renata Schaal, Fang Wang, Tiffany Tsou, Wilson McKerrow, David Keefe

**Affiliations:** 1 Department of Obstetrics and Gynecology, Division of Reproductive Endocrinology and Infertility, New York University Langone Fertility Center, 660 1st Avenue, New York, NY 10016, USAdavid.keefe@nyulangone.org (D.K.); 2 Institute for Systems Genetics, New York University Langone Medical Center, 550 1st Avenue, New York, NY 10016, USA

**Keywords:** oocytes, DMSO, dimethyl sulfoxide, vitrification, germinal vesicle, epigenetics, gene expression

## Abstract

**Simple Summary:**

Alterations in gene expression occur in immature oocytes after vitrification with DMSO-containing cryoprotectant.

**Abstract:**

Despite substantial advancements in the field of cryobiology, oocyte and embryo cryopreservation still compromise developmental competence. Furthermore, dimethyl sulfoxide (DMSO), one of the most commonly used cryoprotectants, has been found to exert potent effects on the epigenetic landscape of cultured human cells, as well as mouse oocytes and embryos. Little is known about its impact on human oocytes. Additionally, few studies investigate the effects of DMSO on transposable elements (TE), the control of which is essential for the maintenance of genomic instability. The objective of this study was to investigate the impact of vitrification with DMSO-containing cryoprotectant on the transcriptome, including on TEs, of human oocytes. Twenty-four oocytes at the GV stage were donated by four healthy women undergoing elective oocyte cryopreservation. Oocytes were paired such that half from each patient were vitrified with DMSO-containing cryoprotectant (Vitrified Cohort), while the other half were snap frozen in phosphate buffer, unexposed to DMSO (Non-Vitrified Cohort). All oocytes underwent RNA sequencing via a method with high fidelity for single cell analysis, and which allows for the analysis of TE expression through Switching Mechanism at the 5′-end of the RNA Transcript sequencing 2 (SMARTseq2), followed by functional enrichment analysis. Of the 27,837 genes identified by SMARTseq2, 7331 (26.3%) were differentially expressed (*p* < 0.05). There was a significant dysregulation of genes involved in chromatin and histone modification. Mitochondrial function, as well as the Wnt, insulin, mTOR, HIPPO, and MAPK signaling pathways were also altered. The expression of TEs was positively correlated with the expression of *PIWIL2*, *DNMT3A*, and *DNMT3B*, and negatively correlated with age. These findings suggest that the current standard process of oocyte vitrification, involving DMSO-containing cryoprotectant, induces significant transcriptome changes, including those involving TEs.

## 1. Introduction

Since the experimental label on oocyte cryopreservation (OC) was lifted in 2012, the use of OC has increased exponentially in the United States and around the world [1,2]. Almost 70,000 autologous and donor OC cycles have been documented in the United States per the Society for Assisted Reproductive Technology database. Unsurprisingly, OC is now considered the first-line option for fertility preservation for medical and elective indications [2]. Advancements in OC techniques have improved the rates of oocyte thaw survival, fertilization, embryo development, and embryo transfer outcomes [1].

Though generally considered innocuous, the short- and long-term safety profiles of OC remain unknown [2]. A paired randomized controlled trial in the US found comparable rates of implantation and aneuploidy, but decreased rates of fertilization and embryo development from vitrified vs. fresh oocytes [3]. Subsequent studies reported lower blastocyst formation and live birth rates from vitrified vs. fresh oocytes [4,5]. Other studies reported a small yet statistically significant increased rate of early childhood cancer in the offspring of various types of assisted reproductive technology (ART) compared to the general population [6,7,8]. Fetal macrosomia, low birth weight, prematurity, birth defects, growth/metabolic disorders, genomic imprinting diseases, and psychomotor/mental developmental delays have also been associated with ART [2,9]. Less clear are which steps in the ART process contribute to these adverse outcomes, their biological basis, as well as whether those outcomes result from ART itself or from underlying parental pathophysiology. 

A central area of concern is the role of ART, including cryopreservation, in the epigenetic landscape during gametogenesis [2]. During the early female fetal period, primordial germ cells (PGCs) undergo genome wide de-methylation, and remain hypo-methylated until the post-pubertal period, when re-methylation takes place during the final stages of oocyte maturation [2,10,11]. Fluxes in the epigenetic state during gametogenesis leave the oocyte particularly vulnerable to disruption from manipulations involved with ART [2]. In most mammalian species, transcription is repressed until the activation of the zygotic genome, a process which depends on the attachment of cumulus cells [12]. This could limit the utility of the transcriptome as a readout of the cell’s epigenetic state, except that during the cryopreservation process cumulus cells are stripped for morphological assessment. 

Dimethyl sulfoxide (DMSO), one of the most widely used cryoprotective agents for oocyte cryopreservation, is a permeating, highly water-soluble cryoprotective agent that is known to impact cell differentiation, apoptosis, ion channel expression, lipid metabolism, and histone deacetylase inhibition [13,14]. DMSO also functions as a methyl donor [13], raising the question of whether it alters cytosine and histone methylation in oocytes, processes which are central to epigenetic regulation. With respect to varying exposure duration and concentration, vitrification with DMSO can negatively impact oocyte and embryo development in bovine, murine, and porcine oocytes [15,16,17]. Additionally, DMSO exerts potent effects on the epigenetic landscape of cultured human cells, including hepatic, cardiac, and embryonic stem cells [18,19]. 

In sum, the literature suggests a propensity towards epigenetic modifications in response to DMSO exposure. Monzo et al. reported the dysregulated expression of 608 genes in 17 fresh and 36 vitrified human oocytes by global microarray analysis [20]. Similarly, Chamayou et al. found dysregulated gene expressions of 17 specific genes involved in DNA structural organization, chromosomal structure maintenance, the energetic pathway, cell cycle regulation processes, and stem cell markers, using reverse transcription PCR to study 15 vitrified and 15 fresh human oocytes. Additionally, Chamayou et al. found that only 63% of mRNA content was maintained after vitrification [21]. 

Less is understood about the impact of vitrification with DMSO on the expression and function of TEs (“jumping genes”) [2,10]. TEs comprise over half of the human genome, and presumably evolved from ancient infections of human ancestral genomes by retroviruses [22,23]. In humans, most TEs have been silenced by truncation and/or epigenetic silencing [23], but 50 to 100 copies of one TE, Long Interspersed Nuclear Element-1 (LINE-1), remain capable of autonomous retrotransposition [24]. During gametogenesis and early embryo development, most epigenetic silencing is reversed, and then is reset at the beginning of life [2]. These changes lead to physiological TE activation, which can impact the genome through insertional mutagenesis, the alteration of splice sites, and the altered activity of enhancers and promoters [22,23,24,25]. One of the most intriguing functions of TEs is the regulation of zygote genomic activation (ZGA), and thus totipotency in the early embryo [26]. 

Despite the central role of epigenetic remodeling during oogenesis, the increasing popularity of OC, the widespread use of DMSO during OC, and DMSO’s known role as a methyl donor, little is known about how vitrification with DMSO exposure impacts the transcriptome of the human oocyte. Standard bioinformatic algorithms for RNAseq typically filter out TEs, but we employed a bioinformatic platform, which enables an analysis of TE expression from a single cell RNAseq to analyze the effects of vitrification with DMSO on TE expression in human oocytes, in addition to the global transcriptome. 

## 2. Materials and Methods

Design: This prospective paired controlled cohort laboratory study was conducted between February and June 2021.

### 2.1. Patients and Oocyte Collection

Twenty-four fresh oocytes at the germinal vesicle stage were donated by four healthy women undergoing elective oocyte cryopreservation. All subjects self-identified as Caucasian and underwent oocyte retrieval following an antagonist-controlled ovarian hyperstimulation protocol. The mean patient age was 31.5 years (range 27–35) and mean body mass index (BMI) was 23.5 kg/m^2^ (range 21–31). The mean serum AMH, baseline FSH, and estradiol levels on the day of trigger were 5.6 ng/mL (range 2.6–8.6), 5.8 mIU/mL (range 4.4–7.6), and 3526 pg/mL (range 2149–4936), respectively. The mean number of days of stimulation was 10.5 (range 9–12) and the mean total number of oocytes retrieved at oocyte retrieval was 27.5 (range 10–39). Each patient donated germinal vesicle stage oocytes (4–8 each) for research, in lieu of discarding them. Oocytes were collected following written consent and with Institutional Review Board approval (Study# i16-00154_CR5). Patient characteristics and cycle parameters were extracted from the electronic medical records. Patient characteristics and cycle parameters are displayed in Table 1.

### 2.2. Oocyte Cryopreservation

To control for patient specific characteristics and allow within-patient comparisons, oocytes were paired such that half of the oocytes from each patient were randomly designated for vitrification with DMSO-containing cryoprotectant (“Vitrified Cohort”). The other half were randomly designated to the Non-Vitrified Cohort, and thus were snap frozen, unexposed to DMSO, at −80 °C.

Vitrification was performed following the Simplified Oocyte Vitrification Protocol from the FUJIFILM Irvine Scientific vitrification system (Santa Ana, CA, USA), which includes DMSO based cryoprotectant. Oocytes to be vitrified were exposed to HEPES buffer, equilibration solution, and vitrification solution, as instructed in the protocol. The equilibration solution was prepared with 7.5% DMSO, 7.5% ethylene glycol, 20% dextran serum supplement (DSS), and gentamicin in M-199 HEPES buffered medium. The vitrification solution was prepared with 15% DMSO, 15% ethylene glycol, 0.5 M sucrose, 20% DDS, and gentamicin in M-199 HEPES buffered medium. One to two oocytes were loaded onto each Cryotip^TM^ at room temperature, and then submerged in liquid nitrogen for storage. This group represents vitrified oocytes (V = “Vitrified Cohort”) in later analyses.

The remaining oocytes were individually stored in polymerase chain reaction (PCR) tubes with 2 μL of phosphate buffer solution, and then stored at −80 °C until analysis. These oocytes, representing the control group/fresh oocytes, were Non-Vitrified and not exposed to DMSO (NV = “Non-Vitrified Cohort” in later analyses). All samples remained in storage until sample collection was completed (1–4 weeks). 

### 2.3. Oocyte Thawing

Vitrified oocytes were thawed following the Warming Oocyte and Embryos Protocol by the FUJIFILM Irvine Scientific vitrification system (Cat# 90183, Santa Ana, CA, USA). Briefly, the Cryolock containing vitrified oocytes was quickly removed from liquid nitrogen and placed into 1 mL of prewarmed thawing solution for 1 min to release oocytes. Then, the vitrified oocytes were transferred into the dilution solution for 4 min followed by incubation in the washing solution twice for a total of 8 min. Individual thawed oocytes and non-vitrified oocytes were completely denuded by incubation in acidic Tyrode’s solution to remove zona pellucidae, and then washed in 0.1% PBS/PVP 3 times before being placed in PCR tubes. All denuded oocytes were stored in a −80 °C freezer until being thawed on ice for amplification.

### 2.4. RNA Sequencing and Gene Analysis

All oocytes underwent RNA sequencing via Switching Mechanism At the 5′-end of the RNA Transcript sequencing 2 (SMARTseq2) (SingulOmics Corporation, New York, NY, USA). This method enables single cell global gene expression profiling, including TEs. In brief, after cell lysis, complementary DNA (cDNA) was synthesized via specialized reverse transcriptase. cDNA amplification and library preparation were performed using the Takara SMART-Seq Single Cell Kit (SSsc) and Nextera XT DNA Library Preparation Kit. To optimize quality and reliability, quality control was performed to remove reads containing adapters, bases that could not be determined >10%, and low quality reads using Fastp v0.23.1.

### 2.5. Statistical Analyses

Multiple analytical software were utilized for data analysis. Genes were mapped to the human reference genome (hg38) using STAR v2.61d, followed by transcript quantification with FeatureCounts v1.5.0-p3. Differential gene expression analysis was performed with DESeq2 v1.26.0 (*p* < 0.05) and compared DEGs among both experimental groups and individual samples. These comparisons were then illustrated using principal component analysis plots, a hierarchical clustering heat map, histogram, and volcano plot. Lastly, functional enrichment analyses of the differentially expressed genes were performed, utilizing multiple biological ontology databases via ClusterProfiler v3.8.1, specifically Gene Ontology, Kyoto Encyclopedia of Genes and Genomes (KEGG), and Human Disease Ontology (padj < 0.05). 

### 2.6. RNA Sequencing

#### 2.6.1. Quality Control Analysis

When analyzed against positive and negative controls, all oocytes exhibited DNA fragments within the expected range and profile. However, in a later analysis, (explained further in Section 3), 3 samples were found to be outliers. Based on this intragroup variability, they were excluded from subsequent statistical analyses.

A total of 9,430,875 to 15,155,014 raw reads were generated from each sample. After filtration, 9,418,226 to 13,674,340 clean reads were obtained from each sample. The mean numbers of raw and clean bases identified from each oocyte were 3.39 G (SE 0.09) and 3.35 G (SE 0.088), respectively. Additional calculations of the error rate, Q20, Q30, and GC content of the clean reads indicated good quality and are displayed in Supplementary Table S1.

#### 2.6.2. Gene Mapping

The mean total mapped reads value was 2.1 × 10^7^ (SE 0.05). Collectively, the oocytes had a mean total mapping rate (reads mapped on genome/total reads × 100) of 94% (SE 0.38). The mean rate of reads that were uniquely mapped was 91.7% (SE 0.39), and the mean rate of reads that had multiple mappings was 3.3% (SE 0.05). The mapping data for each sample are displayed in Supplementary Table S2.

#### 2.6.3. Gene Quantification

Gene expression levels were estimated by the abundance of mapped transcripts. Fragments Per Kilobase of transcript sequence per Million base pairs sequenced (FPKM) was used to measure gene expression levels, thus incorporating sequencing depth and gene length. The distribution of genes at different expression levels is presented in Supplementary Table S3. 

### 2.7. Transposable Element Expression Profiling

Raw data obtained from RNA sequencing were used to analyze TE transcripts. Locus-specific TE expression was identified using the BonaFide-TEseq method. SMART-seq2 uses a template switch oligo (TSO) to identify transcripts that have been fully reverse transcribed. The BonaFide-TEseq method extracts reads with this sequence to identify the transcription start sites that overlap with TEs. This allows for the identification of TEs that are expressed from their own promoter activity rather than incorporated into some other transcript. Reads that include the TSO sequence were mapped to specific TE loci using the Software for Quantifying Interspersed Repeat Expression (SQuIRE) (v0.9.9a-beta, Baltimore, MD, USA). Differentially expressed TEs were identified by DESeq2 (v1.16.1; Bioconductor, Boston, MA, USA) as implemented in the SQuIRE call function. R statistical software (v4.1.12; R Foundation for Statistical Computing, Vienna, Austria) was used for all statistical analyses of differentially expressed TEs.

### 2.8. Real Time Quantitative PCR (RT-qPCR)

cDNA remaining after the completion of SMARTseq2 was used for RT-qPCR to validate the results of selected genes and TEs. For RT-qPCR, glyceraldehyde 3-phosphate dehydrogenase (GAPDH) was used as an internal control, and 0.4 μL of original cDNA as a template in 20 μL of real time PCR reaction with iQ SYBR green mixture (Bio-Rad, Hercules, CA, USA). The relative gene expression level was calculated via the 2^−ΔΔ*C*_T_^ method.

## 3. Results

**Figure 1:**
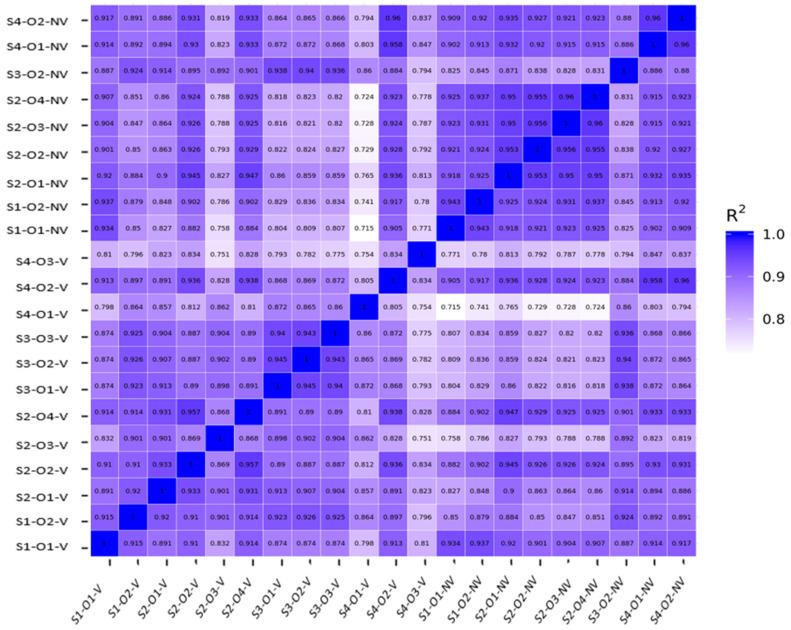
Pearson correlation for gene expression between Vitrified (V) and Non-Vitrified (NV) samples.

**Figure 2:**
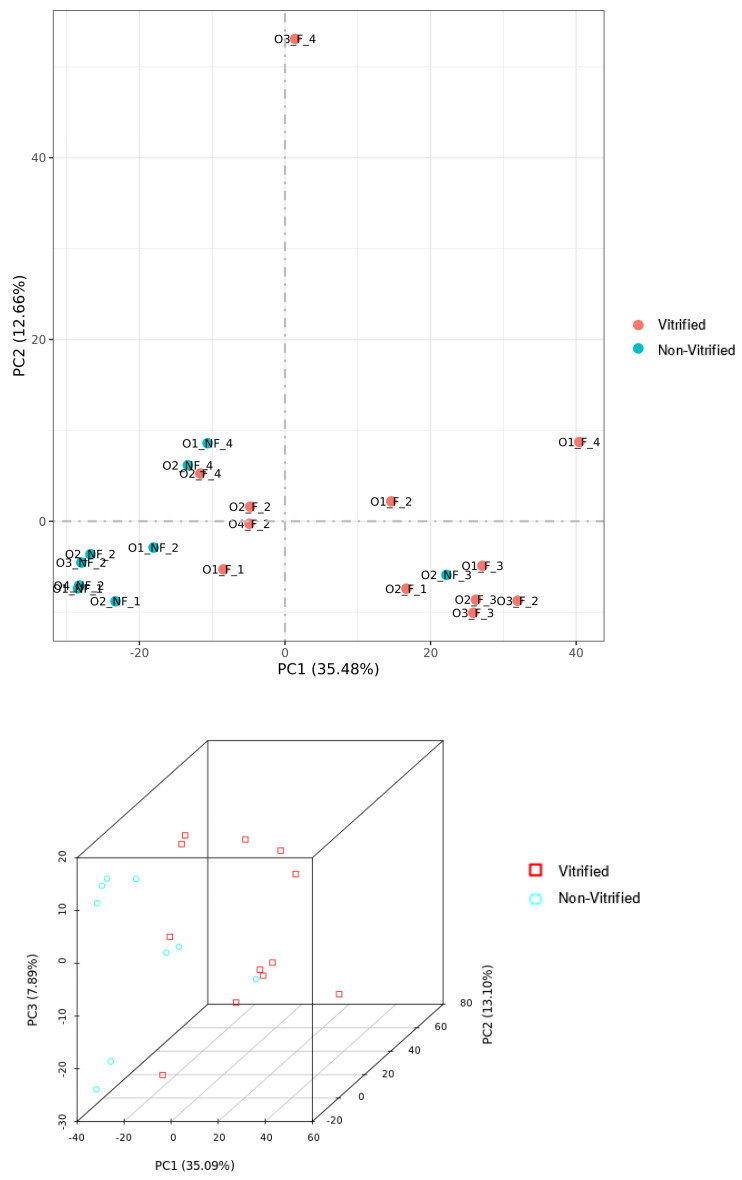
2 and 3-Dimensional principal component analysis (PCA) illustrating intragroup variability among samples.

A total of 11,064 genes with a threshold of >1 FPKM were shared between the V and NV cohorts. Individually, 756 genes were expressed only in the V cohort and 720 genes were expressed only in the NV cohort. Our initial Pearson correlation analysis heat map revealed three outlier samples, all belonging to the NV cohort. Those three outliers were excluded and all analyses were repeated. There was a strong positive correlation among all oocytes, as shown in Figure 1, which is consistent with each group belonging to the same cell type (i.e., intragroup variability). Additional variance among the data set (intergroup variability) was examined by two- and three-dimensional principal component analysis (PCA), illustrated in Figure 2. Plots within the PCA show a general separation of the V vs. NV samples, suggesting distinctions in gene expression (P1: 35.09%. P2: 13.1%, PC3 7.89%, PCA total 56.1%).

### 3.1. Differential Expression Analysis

**Figure 3:**
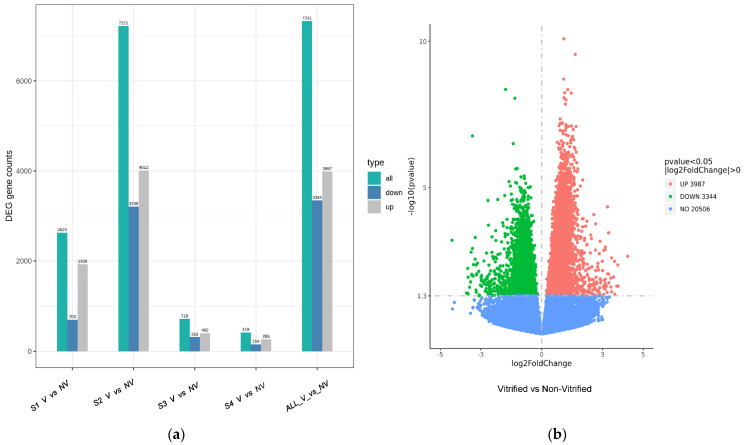
Differential Gene Expression Analysis. (**a**) Histogram shows distribution of all dysregulated, downregulated and upregulated gene expression of samples based on subject and cohort. S: Subject #, V: Vitrified cohort, ND: Non-Vitrified cohort. (**b**) Volcano plot of differential gene expression analysis of all samples on log_2_ fold change scale. The line of significance is at 1.3 log_10_ (*p* value), which corresponds to a *p* value of 0.05 threshold. Red: upregulated genes, Green: downregulated genes, Blue: genes not dysregulated.

A total of 27,837 genes were identified (with no FPKM threshold), of which 7331 (26.3%) were dysregulated. Specifically, the V cohort had 3987 and 3344 upregulated and downregulated DEGs, respectively (>log_2_ fold change, *p* < 0.05), as seen in Figure 3. Further illustration of the pattern of DEGs is shown in hierarchical heat maps, Supplementary Figure S1, analyzed by cohort and subject. A literature review for genes reported to be functionally important for mammalian oocyte developmental competence was performed and individually identified within our data set of DEGs (>log_2_ fold change; *p* < 0.05).

- Genes Upregulated After Vitrification

DEGs that were upregulated after vitrification and associated with *increased* oocyte developmental competence included the following: *Carboxypeptidase D (CPD)*, *SH3 domain binding glutamic acid-rich-like protein (SH3BGRL)*, *High Mobility group AT-hook1 (HMGA1)*, *and G kinase anchoring protein 1 (GKAP1)* [27]. 

DEGs that were upregulated after vitrification and associated with decreased oocyte developmental competence included the following: *Insulin-like growth factor binding protein 3 (IGFBP3)*, *Ndr4 (NDRG4)*, *α thalassemia/mental retardation syndrome X-linked/chromatin remodeler (ATRX)*, *Serine hydroxymethyltransferase 2 (SHMT2)*, *Mediator complex subunit 1 (MED1)*, *Solute carrier family 39 member 14 (SLC39A14)*, *RNA binding motif single stranded interactingprotein 1 (RBMS1)*, *Transforming growth factor β receptor III (TGFBR3)*, *and Mitogen-activated protein 3 kinase 12 (MAP3K12)* [27]. 

Additionally, genes/gene family members involved in transcription regulation, *E2f4* and *Sp3*, which have been associated with decreased oocyte competence [27] were upregulated after vitrification. Intriguingly, *P-element Induced WImpy-2 (PIWIL2)*, a member of the *PIWI* gene family, which is known to be important for preimplantation embryo development, fertility, oocyte production, and TE expression [28], was upregulated after vitrification. Lastly, the epigenetic regulators *DNMT3A* and *DNMT3B* (members of the DNA methyltransferase family) were also upregulated [2] after vitrification.

- Genes Downregulated After Vitrification

A number of DEGs associated with increased developmental competence were downregulated after vitrification, including the following: *Cell division cycle 123 (CDC123)*, *Guanine nucleotide binding protein β 5/G protein subunit β 5 (GNB5)*, *Ubiquitin-conjugating enzyme E2E3 (UBE2E3)*, *Small nuclear ribonucleoprotein D3 (SNRPD3)*, *Ubiquitin-fold modifier 1 (UFM1)*, *and Mitochondrial ribosomal protein L3 (MRPL3)* [27]. 

Some DEGs associated with decreased oocyte developmental competence were downregulated after vitrification, including the following: *Copper transport protein (ATOX1)*, *Fibronectin type 3 and ankyrin repeat domains 1protein (FANK1)*, *Protein transport protein Sec61 subunit γ/Sec61 translocon γ subunit (SEC61G)*, *Calmodulin 2 (CALM2)*, *Basic transcription factor 3-like 4 (BTF3l4)*, *Peroxisomal membrane protein 4 (PXMP4)*, *and Leucine rich repeat containing 28 (LRRC28)* [27].

### 3.2. Functional Enrichment Analyses

- Gene Ontology (GO)

**Figure 4:**
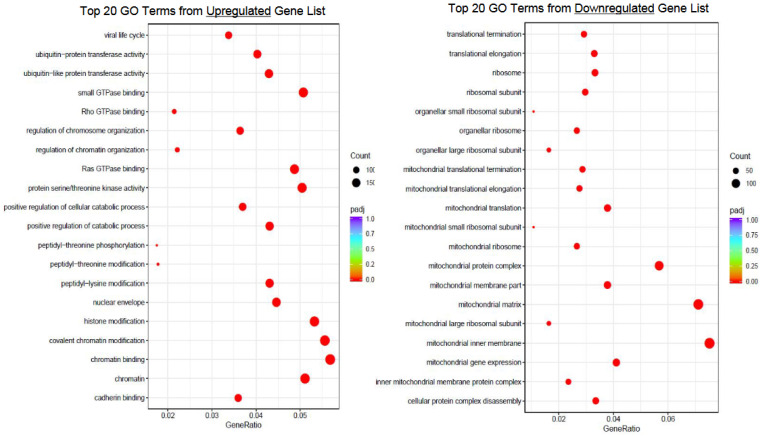
Bubble plot of Gene Ontology (GO) analysis representing the top 20 terms resulting from the upregulated and downregulated gene sets.

The GO terms identified within the upregulated DEGs, after vitrification, included the regulation of chromosome and chromatin organization, covalent chromatin modification, histone modification, and protein serine/threonine kinase activity (padj < 0.05). Among the DEGs downregulated after vitrification were GO terms reflecting multiple mitochondrial components and processes, including mitochondria-associated protein complex, inner membrane, translation, expression, and ribosome (padj < 0.05). The GO analysis results of the top 20 terms are illustrated in Figure 4.

- Kyoto Encyclopedia of Genes and Genomes (KEGG)

**Figure 5:**
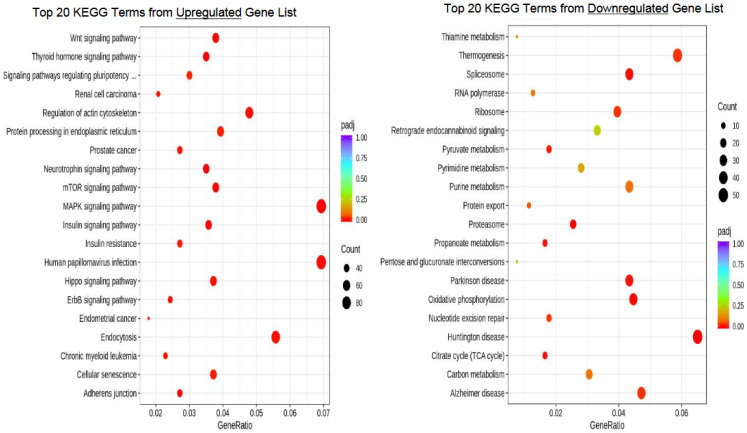
Bubble plot of KEGG (Kyoto Encyclopedia of Genes and Genomes) analysis representing the top 20 terms resulting from the upregulated and downregulated gene sets.

The KEGG terms identified within DEGs upregulated after vitrification included Wnt signaling, signaling pathways regulating pluripotency, mitogen-activated protein kinase (MAPK) signaling, mTOR signaling, insulin signaling, and HIPPO signaling (padj < 0.05). Of DEGs downregulated after vitrification, the KEGG analysis identified terms related to thermogenesis, spliceosome, ribosome, oxidative phosphorylation, and nucleotide excision repair (padj < 0.05). The KEGG analysis results of the top 20 terms are illustrated in Figure 5.

- Human Disease Ontology (HDO)

**Figure 6:**
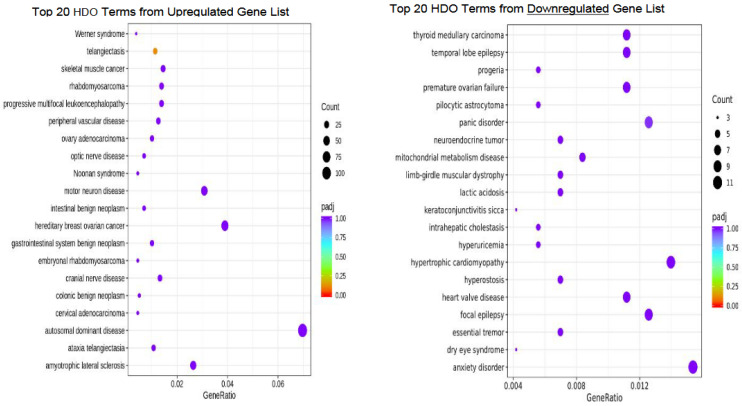
Bubble plot of Human Disease Ontology (HDO) analysis representing the top 20 terms resulting from the upregulated and downregulated gene sets.

There were no significant human disease ontology terms within our data set (Figure 6).

### 3.3. Transposable Element (TE) Expression

A variety of TEs were differentially expressed between the V and NV cohorts, including Alu, endogenous retrovirus family members 1 and K (ERV1, ERVK), and long interspersed nuclear elements 1 (LINE-1). These changes were not consistent across all subjects, but the pattern of TE expression was significantly correlated between oocytes from subjects 2 and 1, as well as between 2 and 4, suggesting that the lack of overlap across all subjects may be due to a lack of statistical power (Supplementary Figure S2). TE expression differed most between the oocytes from subjects 2 and 3, who were the oldest and youngest subjects, respectively (Supplementary Figure S2). Vitrification had the greatest effect on TE expression in the oldest subject (subject 2, age 35 years). Overall, evolutionarily newer and functional TEs were less affected by vitrification, except in oocytes from the oldest patient (Subject 2), which exhibited particular sensitivity. The oocytes from subject 2 exhibited an upregulation of about 5% of all expressed TE loci (Supplementary Figure S3). ERV1 (9%) and ERVK (18%) were particularly upregulated in subject 2’s oocytes after vitrification, with the highest frequency of TE dysregulation seen among the Alu TEs (5%) (Supplementary Figure S3). 

We then sought to determine whether the changes in TE expression after vitrification were associated with changes in the expression of genes involved in the methylation of TE promoters. Notably, *PIWIL2*, *DNMT3A*, *and DNMT3B*, which regulate the epigenetic state of TEs, were upregulated in the V cohort. The expression of TEs was also positively correlated with the expression of *PIWIL2*, *DNMT3A*, and *DNMT3B*. These TEs are considered largely ancient and possibly nonfunctional (Table 2), though we cannot rule out the possibility that they play important roles as promoters or enhancers during early development. Additionally, the upregulation of some TEs was found to be negatively correlated with age (Table 2).

### 3.4. Real Time Quantitative PCR

Six genes/gene families identified by RNA SMARTseq2, as upregulated after vitrification, were selected for further PCR analysis for the validation of the RNA SMARTseq2 results: *DNTM3A*, *DNTM3B*, *ERVK-1*, *ERVK-2*, *ERV1-1*, and *ERV1-2* (Table 3). The upregulation of gene expression in the V cohort was confirmed in all samples.

## 4. Discussion

### 4.1. Differential Gene Expression and Gene Enrichment Analyses

Vitrification with DMSO-containing cryoprotectant alters gene and TE expression in human oocytes. Further studies are needed to confirm whether these changes mediate the known detrimental effects of cryopreservation on oocyte and embryo developmental competence, and/or contribute to long term complications in offspring. In our study, 26.3% of genes within oocytes were perturbed after vitrification. Intriguingly, some of the identified DEGs are known to affect the epigenetic landscape, oocyte maturation, and oocyte developmental competence [2,27,28]. Not all of the effects of vitrification on oocyte gene expression can be presumed to be detrimental. Vitrification upregulated some genes associated with enhanced oocyte developmental competence, and downregulated other genes known to hinder oocyte developmental competence, which may reflect the overall beneficial effects of the inclusion of DMSO cryoprotectant on oocyte survival following vitrification and thawing [29]. 

In the vitrified cohort, there were upregulated genes involved in the regulation of chromosome and chromatin organization, covalent chromatin modification, histone modification, and protein serine/threonine kinase activity—all activities essential to meiosis (Figure 4). Presumably, the upregulation of these cellular functions reflects a protective response mechanism, though further studies are needed to better understand their significance. The GO terms identified among downregulated genes included multiple mitochondrial compartments and processes. Indeed, twelve of the top 20 GO terms from the downregulated gene set are implicated in mitochondrial function (Figure 4). From this, we surmise that the process of vitrification, including the use of DMSO cryoprotectant, has a significant disruptive effect on mitochondrial function. Again, further studies are needed to better understand the impact of the dysregulation of these genes on oocyte function and developmental competence, as well as to further elucidate the specific culpable eliciting factors. 

A number of endocrine and paracrine regulators are instrumental to normal oocyte and embryonic development, and these involve various signaling pathways. Notably, Wnt, insulin, mTOR, HIPPO, and MAPK signaling pathways were altered after vitrification (Figure 5). The Wnt signaling pathway has been implicated in oogenesis through its involvement in steroidogenesis, luteogenesis, and stage-specific folliculogenesis [30,31]. The overactivation of the Wnt signaling pathway alters proliferation and cell differentiation [31]. Furthermore, Wnt signaling maintains genome stability and the appropriate methylation of imprinting control regions, as well as the homeostasis of mouse embryonic stem cells [32]. The findings of alterations in insulin signaling are concerning, as insulin is vital for regulating energy homeostasis, which is crucial for oocyte and embryo development and for the maintenance of oocyte quality with age [33,34]. Insulin signaling pathways also have multiple interconnecting intracellular pathways involved in normal follicular and oocyte function, including mTOR signaling through the regulation of glucose uptake [35]. mTOR signaling, also altered after vitrification, is involved in a variety of cellular functions, including cellular proliferation, growth, organogenesis, sexual differentiation, and epithelial-to-mesenchymal transition signaling [36,37]. Ovarian aging has been linked to mTOR, as well as a number of other female reproductive processes including ovarian reserve, folliculogenesis, oocyte maturation, oocyte aging, and ovarian somatic cell steroidogenesis and proliferation [36,38]. Aberrations in mTOR signaling have been linked to infertility and decreased fertilization after IVF in genetically modified mice [36]. Lastly, MAPK signaling regulates oocyte maturation and pre-implantation embryogenesis, and HIPPO signaling is involved in the maternal–zygotic transition, zygote pluripotency, and trophoblast differentiation [39,40,41].The negative effects of vitrification with DMSO on oocyte attrition, fertilization, and embryo development could involve some or all of the aforementioned pathways [3,4]. 

### 4.2. Differential Transposable Element Expression

Our finding of the dysregulated expression of 26.3% of genes suggests there are substantial effects of vitrification with DMSO on the oocyte’s epigenetic state, consistent with what has been reported in other cell types following DMSO exposure [18,19]. Indeed, we found that there was altered expression of the master epigenetic modifiers DNMT3A and DNMT3B, which regulate DNA methylation. DMSO is known to disrupt methylation, which brings into question whether the upregulation of the DNMT gene family members following vitrification is a compensatory response to DMSO’s widespread disruptive effects on DNA methylation [13]. Alternatively, the upregulation of DNMT3A and DNMT3B could signify genomic hypermethylation, which would potentially reduce transcriptional activity. A topic of continued investigation is the dynamics of the transcriptional activity that occurs throughout oocyte development. Early studies have shown a generalized decline in transcription that occurs toward the end of the oocyte growth phase, and is maintained throughout the transition to meiotic competence [42]. This is evolutionarily possible due to the accumulation of stable mRNA at the beginning of oocyte development, which becomes a reservoir for later use to support oocyte maturation and embryogenesis [43]. Transcriptional activity silencing is thought to begin at the “surrounded nucleolus” GV phase and does not fully revert until fertilization [42].

Complicating this concept of “complete” transcriptional silencing are transcriptome profiling studies that report distinct clustering and unique gene expression within human oocytes in comparison to prior growth, maturation, and follicular stages [12,44]. Furthermore, the transcriptional activity of a human oocyte appears to inversely correlate with the presence of surrounding cumulus cells [12,42,45], an entity that is often removed during the standard oocyte morphological assessment prior to oocyte cryopreservation. Though it is clear that there is a concerted decline in global transcription during oocyte development, it remains feasible that some degree of transcriptional activity is maintained throughout, which is likely vulnerable to environmental conditions.

The upregulation of *PIWIL2* is equally intriguing, as members of the PIWI family have recently been shown to recognize and silence TEs in the female germ line [28]. *PIWI2*, *DNMT3A*, and *DNMT3B* would be expected to suppress TE expression [28], but we observed a positive correlation between TEs and *PIWI2*, *DNMT3A*, and *DNMT3B* expression. Based on studies in somatic cells, showing the loss of TE suppression with advancing age, we expected a positive correlation of age with TE expression [46,47]. However, we found most TEs to be negatively correlated with age. It is important to note that the regulation of TE expression differs fundamentally between germ and somatic cells [47]. Moreover, in our study, the TEs most affected following vitrification were of ancient origin, suggesting they may not have retained their reverse transcriptase and endonuclease activities, and/or they may have been co-opted for gene regulatory activities. The significance of these unexpected effects of vitrification with DMSO on TE expression merits further study, especially as to their functional implication in older women undergoing ART.

Evolutionarily newer and functional TEs were less affected by vitrification in subjects 1 and 3. Notably, the opposite was found in oocytes from the oldest patient (subject 2), which exhibited particular sensitivity. The expression of TEs in subject 2 differed significantly from that of the youngest patient. This finding is intriguing, given that our oldest patient, at age 35, is near the threshold of the typical recommended age for elective OC based on outcome data [48,49]. Notably, a maternal age effect on gene expression also was reported by Steuerwald et al., who showed that the expressions of oocyte genes belonging to major functional categories are negatively influenced by maternal age [50]. As stated above, age also affects the regulation of TEs in somatic cells [46,47]. Future investigations should focus on gaining a better understanding of the maternal age effect on TE expression in oocytes. 

The *ERV* TE family would be particularly interesting to investigate in future studies. The *ERVL* TE, found in our study to be positively correlated with *DNMT3A*, regulates ZGA and cell totipotentiality [26]. An ancient TE, *ERVL*, is shared across multiple species, where it has been co-opted during evolution to regulate these essential processes during early embryo development [26]. The overexpression of the signaling pathways regulating pluripotency, combined with the reported potential impact of *ERVL* on totipotentiality, suggests a mechanism by which vitrification with DMSO could impair the developmental capacity of oocytes and embryos. The dysregulation of various Alus in the V cohort was also observed in our study. However, it is important to consider that Alu TEs have a high loci frequency in the human genome, so their high frequency alone may have contributed to their high rate of dysregulation. Additionally, Alu TEs are rich in euchromatin, which makes them more accessible for analysis than TEs enriched in heterochromatin, such as LINE-1 [51]. 

### 4.3. Strengths and Limitations

This study has a number of strengths and limitations. The strengths include the utilization of oocytes from healthy women, thereby contributing to the generalizability of our results. Additionally, we applied TE profiling in addition to genome wide RNAseq. Most bioinformatic pipelines for RNAseq data filter out signals from TEs. Growing evidence suggests a central role for TEs during oogenesis and early embryo development, so our inclusion of TEs represents a novel contribution to the literature. Additionally, our study design aimed to control for patient-specific variation, which can confound studies of human oocytes. Our paired study design allowed each subject to serve as their own control, decreasing the confounding impact of genetic variability among humans and oocytes. Another strength was the utilization of SMARTseq2 for RNA sequencing, thereby providing a more robust transcriptome profile. A comparative study of six different RNA sequencing methods found that, while not the most cost-effective, SMARTseq2 is currently the most sensitive and accurate method for RNA sequencing, as it is capable of capturing longer RNA sequences and more DNA within a single cell than previous RNA sequencing technologies. Moreover, SMARTseq2 uniquely covers 3′UTRs, which enables a distinction between functional vs. nonfunctional TEs. Thus, when analyzing single cells, as in our study, SMARTseq2 is the best method currently available [52].

The limitations of our study include the use of GV stage oocytes at the time of retrieval. Though mitigated to some degree by our paired randomized study design, their inadequate response to hormonal stimulation could have yielded a sample of GVs at varying developmental sub-phases, which could contribute to transcriptomic differences. A more favorable sample type would have included MII oocytes, the stage when oocytes have the capacity for fertilization, and when they are typically cryopreserved. However, standard clinical practice during elective OC is to vitrify all MII stage oocytes, thus leaving none for experimentation. Mature human oocytes are rarely available for research. Although, from one perspective, the use of only GV oocytes could be considered a limitation, the insights from our study may enhance the understanding of the poor survival of thawed GV oocytes and in vitro maturation. The technical limitations include the brief duration of the cryopreserved state, which may limit the generalizability of our findings. Reassuring in this regard are studies demonstrating that the duration of cryopreservation does not appreciably affect the developmental potential, and therefore presumably the gene expression, of human oocytes [53,54]. Additionally, our control group, representing fresh non-vitrified oocytes, was snap frozen for study logistics, which could have skewed the transcriptome profile from being an exact reflection of fresh oocytes. Lastly, there are multiple inherent factors involved in the process of vitrification that could contribute to the changes found in gene expression, such as exposure to liquid nitrogen, and the overall impact of vitrification on the oocyte cytoskeleton. Other chemical components and cryoprotectants involved in the vitrification system protocol, such as ethylene glycol, trehalose, and dextran, should also be evaluated. Future studies should include additional comparative cohorts, such as non-stimulated GVs or vitrified GV oocytes, without DMSO exposure. Due to cost, sample scarcity, and the paired study design, this study did not include additional arms to evaluate other factors. However, we were able to compare the transcriptomic states reflecting the farthest ends of the spectrum in regard to oocyte processing (i.e., “fresh” vs. vitrified with DMSO exposure). We contend that DMSO coupled with vitrification likely drives the changes in gene expression based on its known biochemical activity and proven impact on a variety of other human and non-human cell types [13,14,15,16,17,18,19]. Future studies are needed to determine the clinical significance of these findings and whether human MII oocytes respond similarly. Future studies should also elucidate the contributions of individual cryopreservation procedure constituents to transcriptome changes.

## 5. Conclusions

In summary, this is the first study to report the effects of vitrification with DMSO on the complete human oocyte transcriptome and on the expression of TEs, profiled by SMARTseq2, the most accurate, albeit most expensive, RNAseq method currently available. Our results suggest that vitrification with DMSO-containing cryoprotectant alters the expression of many genes, including some that are essential to the oocyte’s epigenetic landscape and developmental competence. Vitrification with DMSO may also preferentially alter the expression of TEs in oocytes from older women. Ample research has shown acceptable pregnancy rates following the transfer of embryos from vitrified oocytes. However, OC is known to impair embryo developmental competence, and long-term outcomes of offspring conceived from vitrified oocytes are lacking. Fundamental research demonstrates that DMSO induces epigenetic changes in cultured cells. Given the importance of oogenesis in establishing the epigenetic states of the embryo, fetus, and newborn, and the widespread clinical use of oocyte cryopreservation and the known effects of DMSO on the epigenetic state of cultured mammalian cells, we believe that this topic warrants further investigation. Our results contribute to the overall understanding of the molecular events that occur during the process of oocyte vitrification, a procedure that has increased exponentially around the world.

## Figures and Tables

**Table 1:** Clinical and cycle characteristics for each subject. AMH = anti-mullerian hormone, FSH = follicle stimulating hormone, E2 = serum estradiol.

Patient Clinical and Cycle Characteristics								
Subject	Age (Years)	BMI (kg/m²)	AMH (ng/mL)	FSH (mIU/mL)	E2 on Trigger Day/1 Day Post Trigger (pg/mL)	Days of Stimulation	Oocytes Retrieved/MII	Vitrified/Non-Vitrified
1	30	21	2.6	6.5	2149/2516	9	10/5	2/2
2	35	21	3.3	4.4	4188/4765	11	29/12	4/4
3	27	31	8.2	4.8	4936/6659	12	39/32	3/3
4	34	21	8.6	7.6	2832/4486	10	32/22	3/3

**Table 2:** Table II: Transposable elements with positive and negative correlation to PIWIL2, DNMT3A, DNMT3B and age.

TE Correlation to PIWIL2, DNMT3A, DNTM3B, and Age		
	Positive Correlation	Negative Correlation
**PIWIL2**	Ancient HERVs: LTR41B, MER51A SINE: MamSINE1 DNA TEs: MER102a, Tigger11a, Tigger3d	
**DNMT3A**	Ancient(mammalian) L1s: L1MB8, L1MC2:L1:LINE, L1ME4b:L1:LINE, LTR41B:ERVL:LT MamSINE1, MIR ERV1 LTRs: MER51A DNATEs: Tigger11a, Tigger3d	
**DNMT3B**	Ancient (mammalian) L1s: L1M2a1, L1M5 A DNA TE: MER63C	MLT1J
**Age**	Ancient LINE-1: HAL1ME	HERVs: LTR14B, LTR1C3, LTR7C, MLT1O

**Table 3:** RT-PCR gene primers.

Primer Name	Sequence
ERV1-1-F	TGG ACC TCT CAC AAC ACA AAC T
ERV1-1-R	AGG GGA ATT CCA GTG GGT CT
ERV1-2-F	ACGCTTTACAGCCCTAGACC
ERV1-2-R	GTCGGGAGCAGATTGGGTAA
ERVK-1-F	GGC CAT CAG AGT CTA AAC CAC G
ERVK-1-R	CTG ACT TTC TGG GGG TGG CCG
ERVK-2-F	GGG TAC CTG GCC CCA TAG AT
ERVK-2-R	CAT CAT CCC TTC TTC CTC AGG TT
GAPDH-R	CCC TTT TGG CTC CAC CCT
GAPDH-F	TTC ACC ACC ATG GAG AAG GC
DNMT3A-F	CAA GGA GGA GCG CCA AGA
DNMT3A-R	ACT TGG AGA TCA CCG CAG G
DNMT3B-F	CCC AGC TCT TAC CTT ACC ATC G
DNMT3B-R	GGTCCCCTATTCCAAACTCCT

## Data Availability

The data underlying this article will be shared upon reasonable request to the corresponding authors.

## Supplementary Materials

The following supporting information can be downloaded at https://www.mdpi.com/article/10.3390/genes14061232/s1, Figure S1: Hierarchial clustering map representing diferential gene expression in all samples, analyzed by group and subject. S#: subject #, V = Vitrified cohort; NV = Non-Vitrified cohort. Red to blue gradient color scheme correlates with higher and lower gene expression, respectively. Figure S2: Transposable element expression correlation between Subject 2 and 1; Subject 2 and 4; Subject 2 and 3. Figure S3: Volcano plot illustrating differental expression of transposable elements in samples (Vitrified vs. Non-Vitrified) from Subject 2. The line of siginificance is at 2.2 log10 (*p* value), which corresponds to a false discovery rete (FDR) of 0.05 thresshold and a *p* = 0.0062. Table S1: RNA sequencing data and quality control analyses for each sample. V = Vitrified cohort; NV = Non-Vitrified cohort. Table S2: Summary of reads mapped to human reference genome for each sample. V = Vitrified cohort; NV = Non-Vitrified cohort. Table S3: The distribution of genes at different expression levels for each sample. V = Vitrified cohort; NV = Non-Vitrified cohort.
