# Supplementary material for: Vitrification with Dimethyl Sulfoxide Induces Transcriptomic Alteration of Gene and Transposable Element Expression in Immature Human Oocytes
Source: Genes (Basel). 2023 Jun 8;14(6):1232. doi: 10.3390/genes14061232 (PMC10298107; doi:10.3390/genes14061232)
Supplement: Supplementary file 1 [file genes-14-01232-s001.zip › genes-2392878-supplementary.pdf]

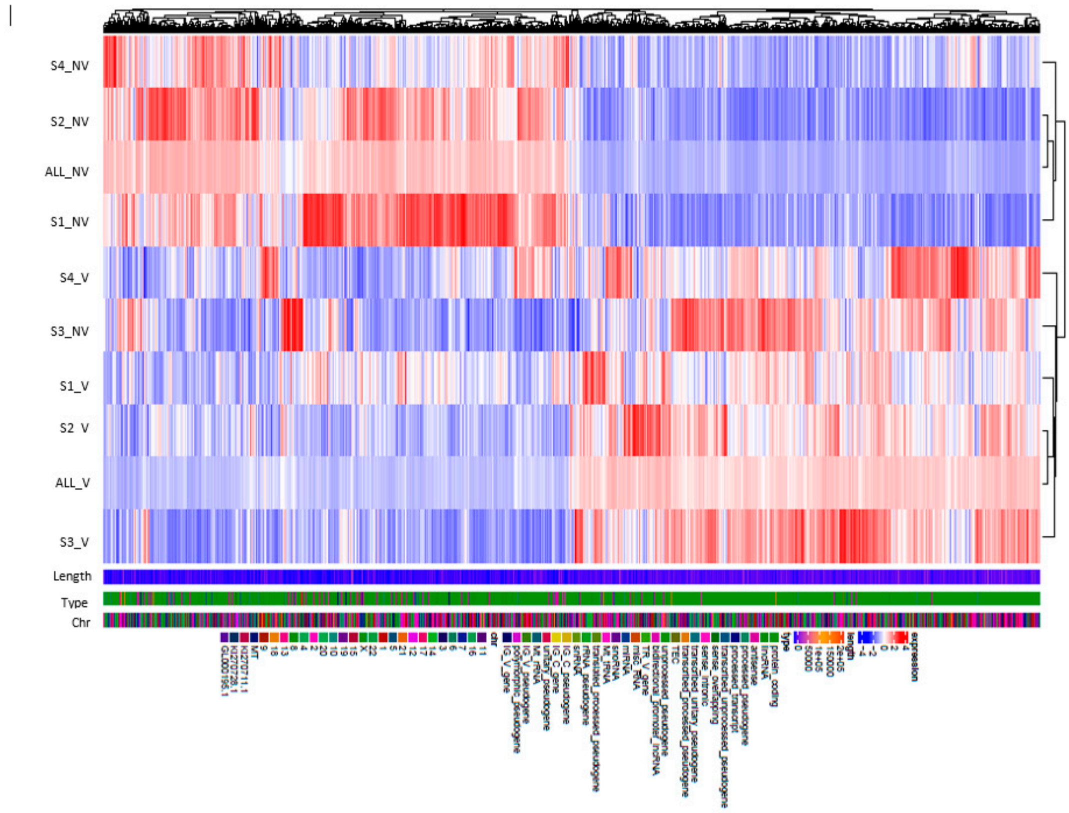

Figure S1: Hierarchial clustering map representing diferential gene expression in all samples, analyzed by group and subject. S#: subject #, V = Vitrified cohort; NV = Non-Vitrified cohort. Red to blue gradient color scheme correlates with higher and lower gene expression, respectively.

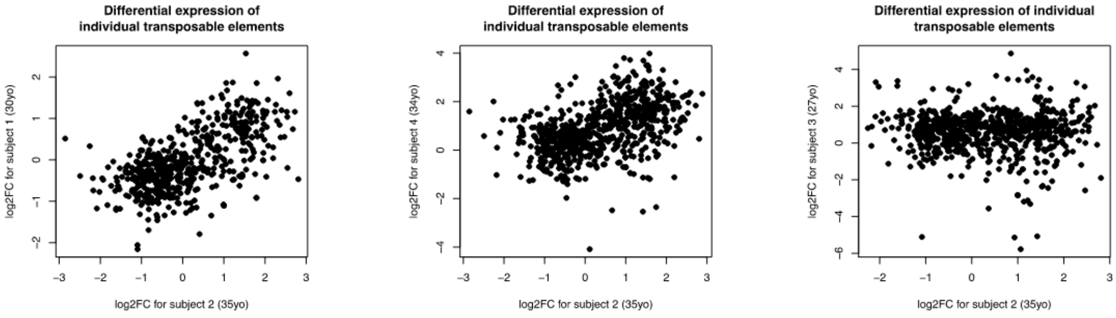

Figure S2: Transposable element expression correlation between Subject 2 and 1; Subject 2 and 4; Subject 2 and 3.

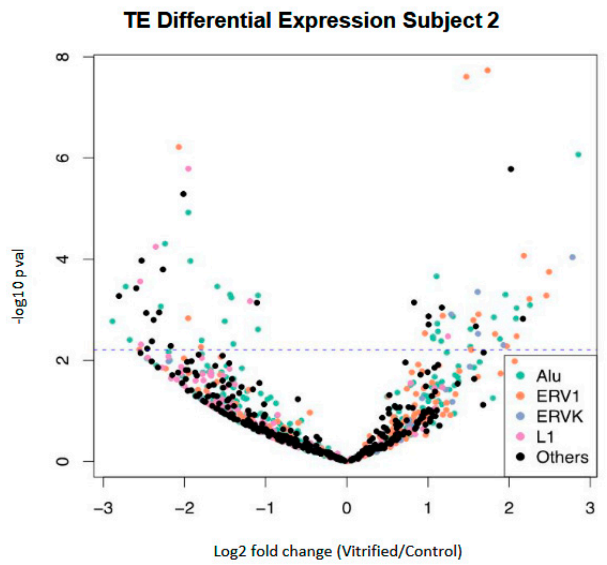

Figure S3: Volcano plot illustrating differental expression of transposable elements in samples (Vitrified vs. Non-Vitrified) from Subject 2. The line of siginificance is at 2.2 log10 (*p* value), which corresponds to a false discovery rete (FDR) of 0.05 thresshold and a $p = 0.0062$.

Quality Control Analysis								
**Sample name**	**Raw reads**	**Clean reads**	**Raw bases**	**Clean bases**	**Error rate (%)**	**Q20 (%)**	**Q30 (%)**	**GC content (%)**
Subject 1-O1-V	11090020	11075275	3.3G	3.3G	0.06	92.28	77.28	46.26
Subject 1-O2-V	9766770	9745809	2.9G	2.9G	0.06	92.21	77.34	47
Subject 1-O1-NV	11060932	9969549	3.3G	3.0G	0.06	92.18	76.93	46.85
Subject 1-O2-NV	10528675	10513396	3.2G	3.2G	0.06	92.24	77.1	47.38
Subject 2-O1-V	10468781	10451683	3.1G	3.1G	0.06	92.31	77.3	46.61
Subject 2-O2-V	10810563	10792750	3.2G	3.2G	0.06	92.43	77.32	48.15
Subject 2-O3-V	11881430	11854919	3.6G	3.6G	0.06	92.24	77.46	46.72
Subject 2-O4-V	11616917	11597862	3.5G	3.5G	0.06	92.39	77.37	48.31
Subject 2-O1-NV	9851521	9838997	3.0G	3.0G	0.06	92.41	77.19	47.95
Subject 2-O2-NV	9430875	9418226	2.8G	2.8G	0.06	92.59	77.6	48.52
Subject 2-O3-NV	11114170	11101213	3.3G	3.3G	0.06	92.53	77.47	48.34
Subject 2-O4-NV	10234518	10222946	3.1G	3.1G	0.06	92.38	77.3	48.26
Subject 3-O1-V	10169006	10150888	3.1G	3.0G	0.06	92.38	77.58	46.71
Subject 3-O2-V	11338221	11318338	3.4G	3.4G	0.06	92.23	77.51	47.37
Subject 3-O3-V	11569711	11547803	3.5G	3.5G	0.06	92.42	77.49	47.37
Subject 3-O2-NV	11481948	11461989	3.4G	3.4G	0.06	92.33	77.53	46.72
Subject 4-O1-V	10402653	10368525	3.1G	3.1G	0.06	91.97	77.7	44.29
Subject 4-O2-V	12635611	12617593	3.8G	3.8G	0.06	92.35	77.42	48.25
Subject 4-O3-V	13022104	13003285	3.9G	3.9G	0.06	92.18	77.05	47.16
Subject 4-O1-NV	15155014	13674340	4.5G	4.1G	0.06	92.2	77.15	48.04
Subject 4-O2-NV	13574842	13553534	4.1G	4.1G	0.06	92.43	77.52	48.27

Supplementary Table S1: RNA sequencing data and quality control analyses for each sample. V= Vitrified cohort; NV= Non-Vitrified cohort

Gene Mapping Analysis							
Sample name	Total reads	Total mapped reads	Uniquely mapped reads	Multiple mapped reads	Total mapping rate	Uniquely mapping rate	Multiple mapping rate
Subject 1-O1-V	22150550	21089652	20416392	673260	95.21%	92.17%	3.04%
Subject 1-O2-V	19491618	18498066	17880928	617138	94.90%	91.74%	3.17%
Subject 1-O1-NV	19939098	18881322	18231778	649544	94.69%	91.44%	3.26%
Subject 1-O2-NV	21026792	20069460	19415528	653932	95.45%	92.34%	3.11%
Subject 2-O1-V	20903366	19812012	19168894	643118	94.78%	91.70%	3.08%
Subject 2-O2-V	21585500	20645712	19962994	682718	95.65%	92.48%	3.16%
Subject 2-O3-V	23709838	22078044	21119342	958702	93.12%	89.07%	4.04%
Subject 2-O4-V	23195724	22280794	21549732	731062	96.06%	92.90%	3.15%
Subject 2-O1-NV	19677994	18888622	18262874	625748	95.99%	92.81%	3.18%
Subject 2-O2-NV	18836452	18090856	17472096	618760	96.04%	92.76%	3.28%
Subject 2-O3-NV	22202426	21355824	20628836	726988	96.19%	92.91%	3.27%
Subject 2-O4-NV	20445892	19681050	19027516	653534	96.26%	93.06%	3.20%
Subject 3-O1-V	20301776	19162806	18476234	686572	94.39%	91.01%	3.38%
Subject 3-O2-V	22636676	21421880	20602474	819406	94.63%	91.01%	3.62%
Subject 3-O3-V	23095606	21745250	20951812	793438	94.15%	90.72%	3.44%
Subject 3-O2-NV	22923978	21621590	20781816	839774	94.32%	90.66%	3.66%
Subject 4-O1-V	20737050	18340580	17651968	688612	88.44%	85.12%	3.32%
Subject 4-O2-V	25235186	24298008	23474336	823672	96.29%	93.02%	3.26%
Subject 4-O3-V	26006570	24966238	24138606	827632	96.00%	92.82%	3.18%
Subject 4-O1-NV	27348680	26264536	25319154	945382	96.04%	92.58%	3.46%
Subject 4-O2-NV	27107068	26025120	25127170	897950	96.01%	92.70%	3.31%

Supplementary Table S2: Summary of reads mapped to human reference genome for each sample. V= Vitrified cohort; NV= Non-Vitrified cohort

Gene Expression Quantification					
FPKM Interval	0--1	1--3	3--15	15--60	>60
Subject 1-O1-V	46965(79.96%)	3885(6.61%)	4882(8.31%)	2095(3.57%)	908(1.55%)
Subject 1-O2-V	46935(79.91%)	3799(6.47%)	5049(8.60%)	2206(3.76%)	746(1.27%)
Subject 1-O1-NV	46977(79.98%)	3826(6.51%)	5022(8.55%)	2247(3.83%)	663(1.13%)
Subject 1-O2-NV	47130(80.24%)	3799(6.47%)	4854(8.26%)	2134(3.63%)	818(1.39%)
Subject 2-O1-V	47063(80.13%)	3739(6.37%)	5007(8.52%)	2207(3.76%)	719(1.22%)
Subject 2-O2-V	47215(80.39%)	3777(6.43%)	4739(8.07%)	2137(3.64%)	867(1.48%)
Subject 2-O3-V	47067(80.13%)	3752(6.39%)	5035(8.57%)	2191(3.73%)	690(1.17%)
Subject 2-O4-V	46890(79.83%)	3853(6.56%)	5124(8.72%)	2174(3.70%)	694(1.18%)
Subject 2-O1-NV	46992(80.01%)	3779(6.43%)	5073(8.64%)	2142(3.65%)	749(1.28%)
Subject 2-O2-NV	47630(81.09%)	3340(5.69%)	4785(8.15%)	2226(3.79%)	754(1.28%)
Subject 2-O3-NV	47269(80.48%)	3728(6.35%)	4619(7.86%)	2150(3.66%)	969(1.65%)
Subject 2-O4-NV	48035(81.78%)	3331(5.67%)	4298(7.32%)	2074(3.53%)	997(1.70%)
Subject 3-O1-V	46866(79.79%)	4005(6.82%)	4643(7.90%)	2110(3.59%)	1111(1.89%)
Subject 3-O2-V	46801(79.68%)	4066(6.92%)	4806(8.18%)	2097(3.57%)	965(1.64%)
Subject 3-O3-V	47141(80.26%)	3825(6.51%)	4678(7.96%)	2142(3.65%)	949(1.62%)
Subject 3-O2-NV	47330(80.58%)	3834(6.53%)	4452(7.58%)	2114(3.60%)	1005(1.71%)
Subject 4-O1-V	47240(80.43%)	3897(6.63%)	4516(7.69%)	2064(3.51%)	1018(1.73%)
Subject 4-O2-V	47147(80.27%)	3935(6.70%)	4532(7.72%)	2122(3.61%)	999(1.70%)
Subject 4-O3-V	47123(80.23%)	3676(6.26%)	4913(8.36%)	2224(3.79%)	799(1.36%)
Subject 4-O1-NV	47319(80.56%)	3683(6.27%)	4650(7.92%)	2177(3.71%)	906(1.54%)
Subject 4-O2-NV	47177(80.32%)	3756(6.39%)	4705(8.01%)	2156(3.67%)	941(1.60%)

Supplementary Table S3: The distribution of genes at different expression levels for each sample. V= Vitrified cohort; NV= Non-Vitrified cohort
